# Decrease of tear break-up time at Japanese eye clinics during five consecutive years

**DOI:** 10.1038/s41598-022-11035-9

**Published:** 2022-04-27

**Authors:** Masahiko Ayaki, Kazuno Negishi

**Affiliations:** 1 Department of Ophthalmology, Keio University School of Medicine, 35 Shinanomachi, Shinjuku-ku, Tokyo, 160-8582 Japan; 2 Otake Clinic Moon View Eye Center, Kanagawa, Japan

**Keywords:** Diseases, Health care, Medical research

## Abstract

The aim of this retrospective chart review study was to evaluate the 5-year trend of ocular surface examination results in participants who visited the eye clinic from 2015 to 2019, underwent corneal and lacrimal examinations, refraction, and intra-ocular pressure measurements, and reported six dry eye-related symptoms. A total of 1468 patients were analyzed. Tear break-up time (BUT) decreased continuously for five consecutive years: 4.76 ± 1.84 s in 2015, 4.57 ± 1.70 s in 2016 (p = 0.999, vs 2015), 4.35 ± 2.06 s in 2017 (p = 0.662), 3.83 ± 2.18 s in 2018 (p < 0.001), and 3.63 ± 2.10 s in 2019 (p < 0.001). The decrease of BUT was more prominent in women than men (p < 0.001) and the correlation coefficient between calendar year and BUT was greater in women than men (p = 0.002). Schirmer test value, strip tear meniscometry value, and corneal staining score did not exhibit significant changes. Prevalence of blurring, photophobia, and pain increased toward 2019 among symptoms surveyed (eye fatigue, blurring, photophobia, dryness, irritation, and pain) and regression analysis indicated blurring (p < 0.001), photophobia (p < 0.001), and pain (p < 0.001) were correlated with BUT. In conclusion, BUT decreased continuously for five consecutive years from 2015 to 2019 and was associated with dry-eye related symptoms.

## Introduction

Dry eye (DE) has been recently defined as a multifactorial disease characterized by a persistently unstable and/or deficient tear film accompanied by variable degrees of ocular surface epitheliopathy, inflammation and neurosensory abnormalities^1^. It causes discomfort and/or visual impairment, and is associated with a variety of comorbidities and decreased quality of life including unhappiness, depression, sleep disorders, asthma, and productivity loss^2–6^. Surveys of DE symptoms and prescription reports^7–9^ suggested DE may be increasing.; diquafosol sodium eyedrop prescription increased every year during 2012–2016 in Japan^7^ and prescriptions of sodium hyaluronate eye drops increased from 2.6 in 2002 to 14.7% in 2015^8^. Ahn et al. reported that the overall prevalence of DE diagnosis and the prevalence of DE symptoms in South Korea were 8.0 and 14.4%, respectively, in 2010–2011 in South Korea^9^.

DE may vary due to seasonality and other factors^10^. Pollen in the air, weather changes, and indoor environmental pollen have been suggested as potential contributory factors for increased prevalence of DE in surveys, coding database, and clinical experiments^11–13^. These studies investigated short-term exposure to adverse indoor environmental conditions. Specifically, air pollution and bioaerosols and dry indoor air caused by air conditioners or fan heaters may be associated with DE. Digital eye strain and increasing screen time on computers and smartphones may be contribute since numerous investigators identified it as a serious issue for DE^14–16^. However, examination results from longitudinal observation across multiple calendar years have not been documented.

The definitions of DE by Asian and other societies^1,17^ emphasized the importance of tear film instability clinically presented as shortening (≤ 5 s) of tear film break-up time (short BUT). Short BUT has been reported in the majority of DED patients in numerous clinical studies^18,19^ indicating that tear film instability measured through BUT is a common feature of DE. The tear film is comprised of a lipid layer, aqueous layer, and mucins of the ocular surface epithelium with unique break-up patterns, and tear film instability may be caused by any of the layers. The recommendation for treatment strategy is based on the diagnosis of subtypes of DE classified by break-up pattern^20^. The aim of this study was to assess 5-year trend of DE-related signs and symptoms, rather than diagnosed DE, in a long-term cohort undergoing comprehensive ophthalmological examinations at eye clinics. In particular, we focused on tear break-up time (BUT) as a representative clinical indicator of DE. Additionally, we selected six common symptoms of DE and asked patients if they experienced them or not. The present study terminated before the COVID-19 pandemic, which may additionally affect systemic health and the status of DE patients due to alterations in lifestyle and mental status^16^.

## Methods

### Study participants and clinical review board approval

The present study was conducted at three eye clinics, A, B, and C located on the Japanese island of Honshu. A and B are located in the eastern region and C is in the central area. This study was a retrospective chart review of routine examinations performed for patients suspected of having DE. The study was approved by the clinical review board and ethics committee of the Tsukuba Central Hospital (approved on 12 December 2014, permission number 141201). The other clinics were approved as collaborating institutes of Tsukuba Central Hospital. This study was carried out in accordance with the Declaration of Helsinki. The need for consent was waived off by the Clinical Review Board of the Tsukuba Central Hospital. The Institutional Review Board and Ethics Committee of Keio University School of Medicine approved this study (approval date: 28 June 2021; approval number 20210080) to permit authorship for authors (KN and MA) who are appointed at the Keio University School of Medicine.

### Inclusion and exclusion criteria

The study consecutively recruited patients from the participating clinics between January 2015 and December 2019. We included outpatients only at first visit with best-corrected visual acuity above 20/30 in both eyes. The exclusion criteria were use of eyedrops, ocular surgery within the previous month, and acute eye disease within the previous week.

### Ophthalmological examinations

The ophthalmological examination was carried out according to Yokoi et al.^21,22^. Briefly, BUT was defined as the time interval between blink and the appearance of the first dark spot in the cornea. From 2016, break-up pattern was classified as ‘line’, ‘spot’, ‘area’, ‘dimple’, and ‘random’ according to Yokoi’s classification^21^. A corneal staining score was determined to grade corneal epitheliopathy, graded at 0–2 for severity and area. Schirmer test was performed without topical anesthesia. Strips of filter paper (Whatman No. 41; Showa Yakuhin Kako, Tokyo, Japan) were placed at the temporal lower conjunctival fornix for 5 min. The length of the wetted filter paper was recorded. Schirmer test was performed at least 5 min after tear strip meniscometry. To measure aqueous availability at the lower tear meniscus, the wetted length for tear strip meniscometry was obtained. The strips (SMTube, Echo Electricity Co., Ltd., Fukushima, Japan) were inserted into the lateral side of the tear meniscus of the lower lid for 5 s without touching the ocular surface. Intra-ocular pressure and refraction were determined by three successive readings with a noncontact tonometer and refractometer (Tonoref™ II, Nidek Co., Ltd., Aichi, Japan). During examination, temperature and humidity of the examination room were adjusted to 21–25 °C and 40–60%, respectively. According to the Japan Meteorological Agency^23^, the mean annual temperature in the city of Tokyo (on the east of Honshu Island) in 2015/2016/2017/2018/2019 was 16.4/16.4/15.8/16.8/16.5 °C and humidity was 68/69/68/70/70%, respectively. Regarding air pollution, a decrease of NO_2_, suspended particulate matter (SPM), SO_2_, CO and an increase of O_3_ were registered during 2000–2010 according to data obtained from the Environmental Restoration and Conservation Agency^24^.

### Patient interviews for dry-eye related symptoms

Patients were asked about the presence or absence of six common DE-related symptoms: dryness, irritation, pain, eye fatigue, blurring and photophobia. These symptoms were selected from validated questionnaire DEQS^25^ as the six most prevalent symptoms reported by outpatients visiting the dry eye clinic of Keio University Hospital in 2012.

### Statistical analysis

The sample size was calculated with a 0.05 margin of error and 95% confidence interval using G*power 3.1 (American Statistical Association). Effect size was derived from a measured value in the current study. An effect size of 0.272 was identified in BUT with an appropriate total sample size of 137 for the comparison of 2015 and 2019. Where appropriate, data are given as the mean ± SD. The data from the right eye for BUT, corneal staining score, Schirmer test value, and tear strip meniscometry value were analyzed. One-way ANOVA and Mann–Whitney U test were performed as appropriate to assess the 5-year variance of parameters. The Bonferroni test was applied to avoid type I errors from multiple comparisons. The regression line was computed for BUT and year by the least-square method and the difference in slopes and correlation coefficient was compared by t-test after stratification for sex. Correlations between signs and symptoms were evaluated using a standardized partial regression coefficient. The number of participants in 2017 and 2018 was low due to a transfer of the investigator (MA). Nevertheless, sample size is still sufficient for statistical analysis and multiple comparison was performed to minimize errors. All analyses were performed using StatFlex (Atech, Osaka, Japan) with p < 0.05 being considered statistically significant.

## Results

**Figure 1:**
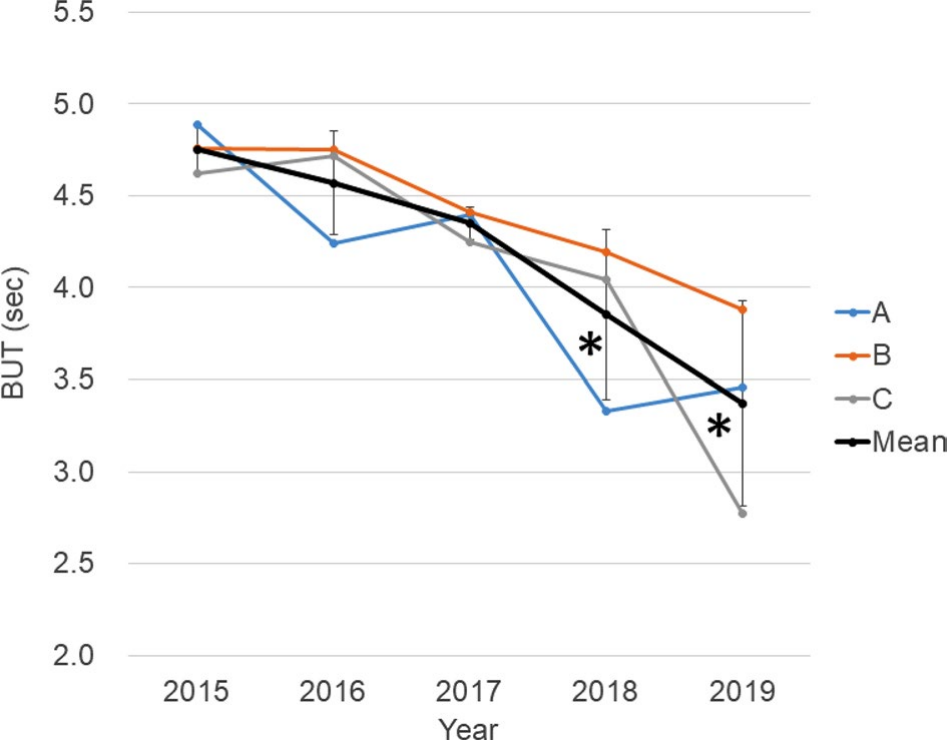
Five-year trend of tear break-up time (BUT) at three clinics (A–C). BUT continuously decreased from 2015 to 1919, and this trend was stable across the three participating clinics. Mean BUT in 2018 and 2019 (black symbol, black line) was shorter than in 2015 (*p < 0.001, vs 2015, Mann–Whitney U test with Bonferroni correction).

**Figure 2:**
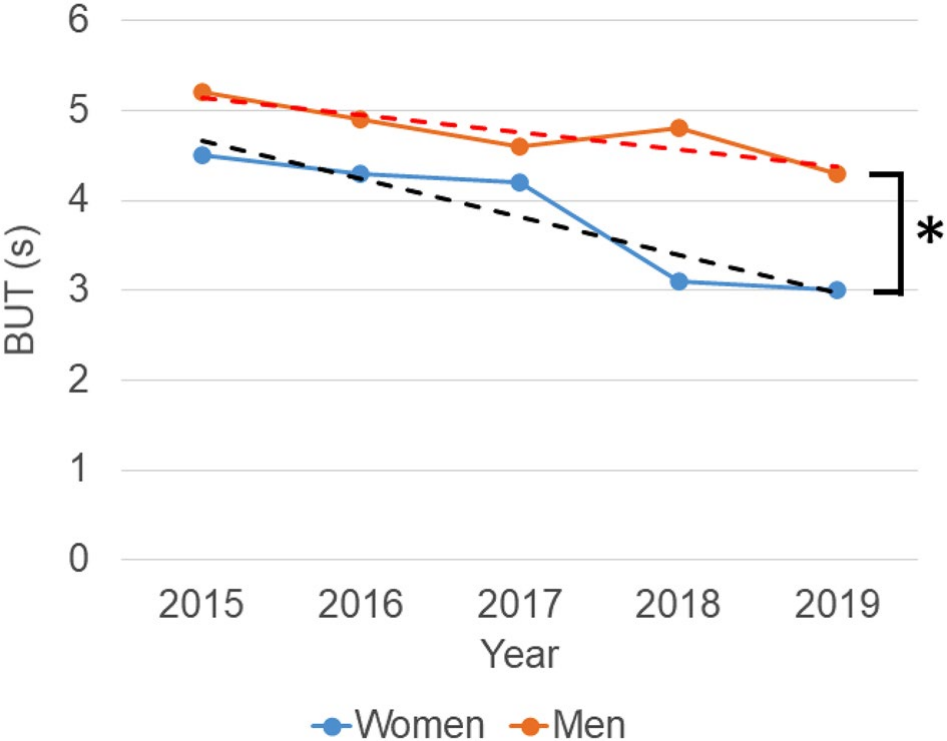
Tear break-up time in each calendar year stratified for sex and regression lines. The decrease of BUT was more prominent in women (blue symbol, blue regression line) compared with men (red symbol, red regression line) (*p < 0.001, regression analysis).

A total of 1468 participants (mean age 59.0 ± 16.5 year, 44.3% male) were analyzed. The participants included 224 individuals (14.3%) with diabetes and three (0.002%) with Sjögren disease. The number of participants per year was 425 (2015), 446 (2016), 123 (2017), 171 (2018), and 303 (2019), and the number of participants per clinic was 479 (A), 557 (B), and 432 (C), respectively. Refraction and intra-ocular pressure did not reveal a specific trend (Table 1). BUT continuously decreased for five consecutive years; 4.76 ± 1.84 s in 2015, 4.57 ± 1.70 s in 2016 (p = 0.999, vs 2015, Mann–Whitney U test with Bonferroni correction), 4.35 ± 2.06 s in 2017 (p = 0.662), 3.83 ± 2.18 s in 2018 (p < 0.001), and 3.63 ± 2.10 s in 2019 (p < 0.001). This trend was stable across the three participating clinics (Fig. 1). The decrease of BUT was more prominent in women than men (p < 0.001) (Fig. 2) and correlation coefficient between year and BUT was greater in women (− 0.291) than men (− 0.137) (p = 0.002).

**Table 1:** Patient characteristics in each calendar year.

Year and parameters	2015	2016	2017	2018	2019	One-way ANOVA
Age (year)	59.9 ± 16.4	54.8 ± 21.0	58.3 ± 16.3	58.9 ± 16.8	62.9 ± 16.6	
Sex (% men)	37.4	50.4	45.1	42.7	46.9	
Refraction (D)	− 1.54 ± 2.95	− 1.75 ± 3.09	− 2.25 ± 3.29	− 1.59 ± 2.93	− 1.70 ± 3.09	
Intraocular pressure (mmHg)	14.27 ± 3.01	14.59 ± 3.23	15.42 ± 3.29	14.90 ± 3.74	14.69 ± 4.25	
Tear break-up time (s)	4.76 ± 1.84	4.57 ± 1.70 (0.999)	4.35 ± 2.06 (0.662)	3.83 ± 2.18 (< 0.001*)	3.63 ± 2.10 (< 0.001*)	F = 21.143, P < 0.001*
Schirmer test (mm)	14.73 ± 10.64	14.52 ± 8.26 (0.999)	16.36 ± 9.74 (0.999)	16.32 ± 10.62 (0.999)	15.27 ± 9.95 (0.999)	F = 0.267, P = 0.899
Tear strip meniscometry (mm)	3.08 ± 2.29	2.85 ± 1.57 (0.999)	2.82 ± 1.94 (0.999)	2.36 ± 1.28 (0.999)	2.52 ± 1.90 (0.999)	F = 0.374, P = 0.826
Corneal staining score	0.31 ± 0.61	0.16 ± 0.46 (< 0.001*)	0.10 ± 0.33 (< 0.001*)	0.20 ± 0.51 (0.055)	0.35 ± 0.60 (0.416)	F = 8.370, P < 0.001*

*p < 0.05, p-value in brackets, vs 2015, Mann Whitney U test with Bonferroni correction.

**Figure 3:**
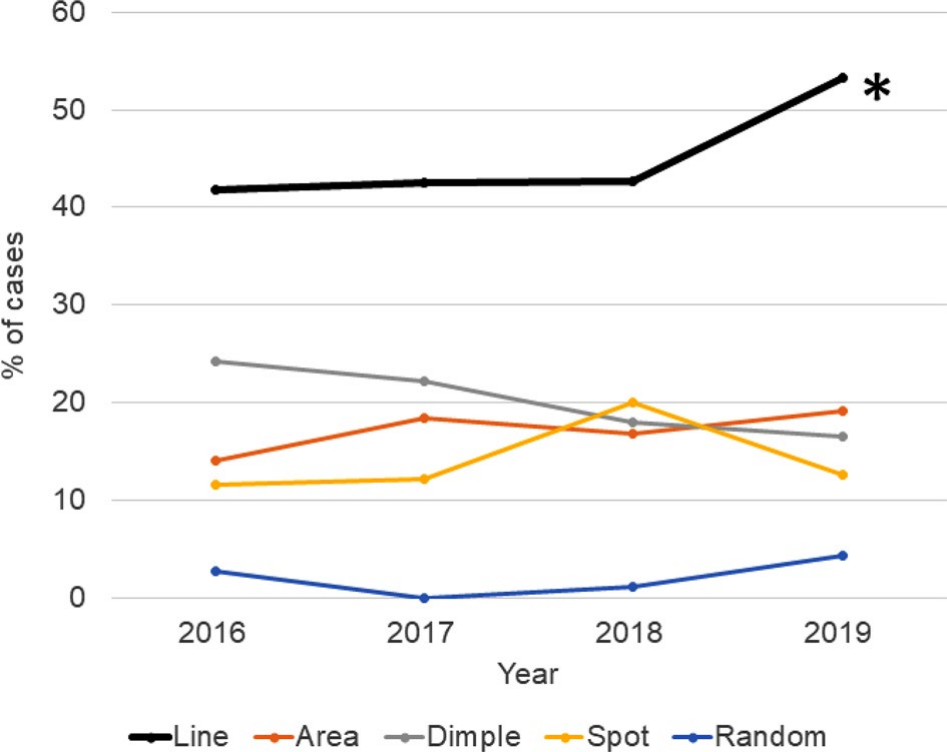
The proportion of tear break-up pattern in each calendar year. Line break pattern was most prevalent and greater in 2019 than 2016 (*p  = 0.026, chi square test).

The line break pattern was most prevalent among five tear break-up patterns and its proportion increased toward 2019 with statistical significance (p = 0.026, 2016 vs 2019, chi square test) (Fig. 3). Tear break-up patterns from 2016 to 2019 could be categorized as line (45.1%), dimple (20.2%), random (2.1%), spot (15.4%), and area (17.2%). Schirmer test value, tear strip meniscometry value, and corneal staining score did not change (Table 1).

**Figure 4:**
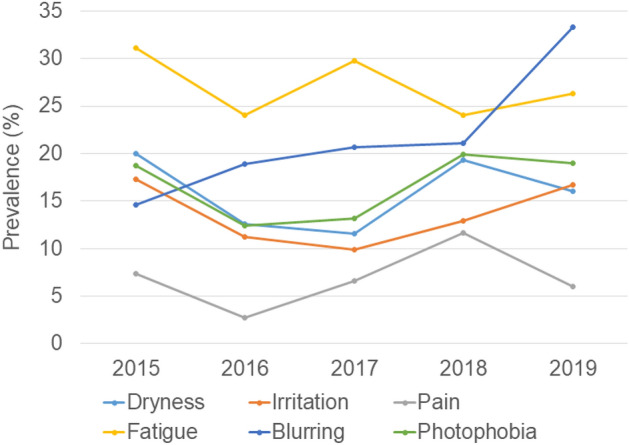
The prevalence of dry eye-related symptoms in each calendar year. Prevalence of blurring continuously increased toward 2019.

The prevalence of blurring increased toward 2019 (Fig. 4). Regression analysis with calendar year and ocular parameter demonstrated that BUT (p < 0.001), corneal staining score (p = 0.008), blurring (p < 0.001), pain (p = 0.030), and photophobia (p = 0.042) were correlated with the calendar year (Table 2). Regression analysis with signs and symptoms revealed that blurring (p < 0.001), photophobia (p < 0.001), and pain (p < 0.001) were correlated with BUT, and pain (p < 0.001) was correlated with corneal staining score (Table 3).

**Table 2:** Correlation between calendar year and ocular parameters.

Variable	Linear regression		Adjusted for age and sex	
	β	*p-*value	β	*p-*value
**Model 1: examination results**				
Tear break-up time (s)	− 0.228	< 0.001*	− 0.233	< 0.001*
Schirmer test (mm)	0.030	0.632	0.105	0.110
Tear strip meniscometry (mm)	− 0.117	0.257	− 0.105	0.304
Corneal staining score	0.069	0.008*	0.066	0.011*
**Model 2: symptoms**				
Dryness	0.020	0.435	0.027	0.290
Irritation	0.022	0.384	0.018	0.489
Pain	0.056	0.030*	0.067	0.010*
Fatigue	− 0.024	0.357	− 0.019	0.452
Blurring	0.132	< 0.001*	0.120	< 0.001*
Photophobia	0.053	0.042*	0.056	0.031*

Calendar years 2015, 2016, and 2017 = 0, 2018 and 2019 = 1.

**Table 3:** Correlation between dry-eye related signs and symptoms.

Variable	Blurring		Photophobia		Pain	
	β	*p*-value	Β	*p*-value	β	*p*-value
Tear break-up time	− 0.085	0.001*	− 0.093	< 0.001*	− 0.120	< 0.001*
Line break type	− 0.016	0.709	0.057	0.195	− 0.001	0.970
Corneal staining score	0.038	0.141	0.042	0.110	0.128	< 0.001*

*P < 0.05, standardized partial regression coefficient, adjusted for age and sex.

## Discussion

The results revealed a continuous decrease of the BUT, which could imply an increasing prevalence of DE throughout the five calendar years. This trend was not limited to specific local clinics and could be generalized nationwide in Japan. Increasing prescription of mucin secretagogue eyedrops (diquafosol sodium) reported by Japan’s largest pharmaceutical company^9^ reasonably supported the current results.

The association of DE and weather and air quality is an emerging issue. As indicated in the “Methods” section, temperature/humidity did not exhibit specific trends during the study period and were not obviously associated with the decrease of BUT. Although rough trends could be inferred from the data on air quality (NO_2_, SPM, SO_2_, CO, and O_3_) from the 1970s to 2010 shown in reference 20, precise values are not available for the study period and correlation analysis was not performed. Additionally, all reported values were below environmental standards issued by the Japanese Ministry of Environment: 0.06 ppm for NO_2_, 0.1 mg/m^3^ for suspended particulate matter, 0.04 ppm for SO_2_, 10 ppm for CO, and 0.06 ppm for O_3_, and the impact of air quality may not be significant during the study period. Moreover, the participating clinics are all located on Honshu Island where the atmosphere is similar. Taken together, environmental factors are unlikely to account for changes in BUT although increased O_3_ and indoor air conditioning remain as potential factors.

Digital eye strain is a serious health problem in the modern computerized society with a growing habitual screen time. According to a cohort study involving 672 Japanese office workers who used visual display terminals, the prevalence of short BUT (≦ 5 s) was as high as 78.6% (mean age: 43.2 years)^26^. A systematic review and meta-analysis of DE in visual display workers estimated the prevalence at 9.5–87.5% depending on criteria of DE^27^. Digital tasks at near distance pose a risk for the development and worsening of DE due to exposure to blue light emitted from the display^28^ and decreased blinking^29^. Another potential hazard is stress. A national survey reported that stress continuously increased in Japanese people from 2013 to 2018^30^, and is a considerable risk factor for DE^31^. In addition, sleep quality is deteriorating with Japanese reporting a mean sleep duration of 7 h 27 m in 1995 that continuously decreased to 7 h 15 m in 2015^32^. Sleep habits could be associated with DE since poor sleep and a disturbed circadian rhythm may worsen DE^33^. It is noteworthy that BUT decreased more rapidly in women than men. The prevalence of DE is much higher in women than men and our results could be a manifestation of women being at higher risk of changes in BUT during this period.

Blurring was strongly associated with BUT and this finding is consistent with numerous previous investigations describing decreased visual function with DE^34^. Pain and photophobia were also correlated with BUT and calendar year. These symptoms are associated with trigeminal neuralgia and could be exacerbated by local or systemic disorders since the prevalence of women’s trigeminal neuralgia is twice that of men^35^, and can be evoked by various triggers including digital eye strain and DE^36^. Synergic effects of DE, digital eye strain, and stress could be responsible for increased blurring, pain, and photophobia during 2015–2019. The present results of the proportion of break-up patterns are comparable to previous results of a multicenter study by Shigeyasu et al.^22^ reporting line (51.6%), dimple (16.3%), random (13.5%), spot (10.5%), and area (4.6%). The line break pattern is observed in the lower cornea of eyes with a mild-to-moderate aqueous tear deficiency. This may be related to a temporary decrease of the aqueous tear thickness in that region as the lower meniscus takes up moisture and an upward drag caused by the tear film spreading^21^. In the present study, Schirmer test values and strip meniscometry values did not change for 5 years and their trend did not correspond to the trend of line break patterns, hence, we speculate that an impaired moisturizing function of corneal epithelial cells and mucin or oil layer dysfunction rather than aqueous tear deficiency may be responsible for an increased proportion of line break patterns.

Strengths of the current study include being a multicenter study, having sufficient sample size, and accumulating 5 years of data from physician examinations. This study measured the prevalence and severity of ocular status as well as DE-related symptoms in a clinic-based cohort. Participating clinics were distributed across the center and east of Japan’s Honshu Island and the results could be generalized.

The current study has several limitations. Although a detailed evaluation of the symptoms was conducted in the present study, dry eye questionnaires including OSDI and DEQ-5 that can quantitatively evaluate symptoms were not applied. This lack may be an insufficiency in this study and should be ameliorated in follow-up research. An analysis of the role of psychological status, lifestyle, other systemic comorbidities, and medications in addition to local environmental factors would enhance the results of the current study although we consecutively recruited first-visit patients only and the participants may be representative of the general population including individuals with adequate systemic health. It is also recommended that these factors (or their proxies VDT use time, anxiety index and average sleeping time) should be recorded and evaluated in a subsequent study.

In conclusion, BUT continuously decreased as the prevalence of blurring, pain and photophobia increased at Japanese eye clinics from 2015 to 2019. Decrease of BUT was more prominent in women than men. We hypothesize that DE and digital eye strain due to rapid digitalization and a decreasing sleep duration and increasing stress may synergistically contribute to the findings.

## Abbreviations

DE: Dry eye
BUT: Tear break-up time

## References

[1] Tsubota K (2020). Defining dry eye from a clinical perspective. Int. J. Mol. Sci..

[2] Kawashima M (2020). Association of systemic comorbidities with dry eye disease. J. Clin. Med..

[3] Ayaki M (2018). Sleep disorders are a prevalent and serious comorbidity in dry eye. Investig. Ophthalmol. Vis. Sci..

[4] Kitazawa M (2018). The relationship of dry eye disease with depression and anxiety: A naturalistic observational study. Transl. Vis. Sci. Technol..

[5] Huang YC (2018). Association between dry eye disease and asthma: A nationwide population-based study. Peer J..

[6] Rakofsky JJ, Rakofsky SI, Dunlop BW (2021). Dry those crying eyes: The role of depression and antidepressants in dry eye disease. J. Clin. Psychopharmacol..

[7] Ahn JM (2014). Prevalence of and risk factors associated with dry eye: The Korea National Health and Nutrition Examination Survey 2010–2011. Am. J. Ophthalmol..

[8] Son K-B (2020). Trends in the utilization of sodium hyaluronate eye drops, including disposable and multiuse forms, in South Korea: A 14-year longitudinal retrospective cohort study. Front. Pharmacol..

[9] Annual report of Santen Pharmaceutical Co. Ltd. www.santen.co.jp (Accessed 15 October 2021).

[10] Ayaki M (2017). Possible association between subtypes of dry eye disease and seasonal variation. Clin. Ophthalmol..

[11] Zhong J-Y (2018). Association between dry eye disease, air pollution and weather changes in Taiwan. Int. J. Environ. Res. Public Health..

[12] Idarraga MA (2020). Relationships between short-term exposure to an indoor environment and dry eye (DE) symptoms. J. Clin. Med..

[13] Galor A, Kumar N, Feuer W, Lee DJ (2014). Environmental factors affect the risk of dry eye syndrome in a United States veteran population. Ophthalmology.

[14] Sheppard AL, Wolffsohn JS (2018). Digital eye strain: Prevalence, measurement and amelioration. BMJ Open Ophthalmol..

[15] Jaiswal S (2019). Ocular and visual discomfort associated with smartphones, tablets and computers: What we do and do not know. Clin. Exp. Optom..

[16] Zhong B (2020). Association of social media use with mental health conditions of nonpatients during the COVID-19 outbreak: Insights from a National Survey Study. J. Med. Internet Res..

[17] Tsubota K (2017). New perspectives on dry eye definition and diagnosis: A consensus report by the Asia dry eye society. Ocul. Surf..

[18] Kawashima M (2017). A clinic-based survey of clinical characteristics and practice pattern of dry eye in Japan. Adv. Ther..

[19] Uchino M (2013). Prevalence of dry eye disease and its risk factors in visual display terminal users: The Osaka study. Am. J. Ophthalmol..

[20] Yokoi N, Georgiev GA (2019). Tear-film-oriented diagnosis for dry eye. Jpn. J. Ophthalmol..

[21] Yokoi N (2017). Classification of fluorescein breakup patterns: A novel method of differential diagnosis for dry eye. Am. J. Ophthalmol..

[22] Shigeyasu C (2020). Characteristics and utility of fluorescein breakup patterns among dry eyes in clinic-based settings. Diagnostics (Basel).

[23] Japan Meteorological Agency. www.jma.go.jp (Accessed 13 October 2021).

[24] Environmental Restoration and Conservation Agency. https://www.erca.go.jp/ (Accessed 13 May 2021).

[25] Sakane Y (2013). Development and validation of the dry eye-related quality-of-life score questionnaire. JAMA Ophthalmol..

[26] Yokoi N (2015). Importance of tear film instability in dry eye disease in office workers using visual display terminals: The Osaka study. Am. J. Ophthalmol..

[27] Courtin R (2016). Prevalence of dry eye disease in visual display terminal workers: A systematic review and meta-analysis. BMJ Open.

[28] Marek V (2018). Blue light phototoxicity toward human corneal and conjunctival epithelial cells in basal and hyperosmolar conditions. Free Radic. Biol. Med..

[29] Golebiowski B (2020). Smartphone use and effects on tear film, blinking and binocular vision. Curr Eye Res..

[30] Japanese Ministry of Health and Labor. *National Survey Report on Stress 2013–2018*. h30-46-50_kekka-gaiyo02.pdf. www.mhlw.go.jp (Accessed 13 October 2021).

[31] Wang MT (2021). Association between dry eye disease, self-perceived health status, and self-reported psychological stress burden. Clin. Exp. Optom..

[32] Japan Broadcasting Culture Research Institute. *Survey 2015*. https://www.nhk.or.jp/bunken/research/yoron/index.html (Accessed 13 October 2021).

[33] Hanyuda A (2001). Relationship between unhealthy sleep status and dry eye symptoms in a Japanese population: The JPHC-NEXT study. Ocul. Surf..

[34] Goto E (2006). Optical aberrations and visual disturbances associated with dry eye. Ocul. Surf..

[35] Katusic S (1990). Incidence and clinical features of trigeminal neuralgia, Rochester, Minnesota, 1945–1984. Ann. Neurol..

[36] Rosenthal P, Borsook D (2016). Ocular neuropathic pain. Br. J. Ophthalmol..

